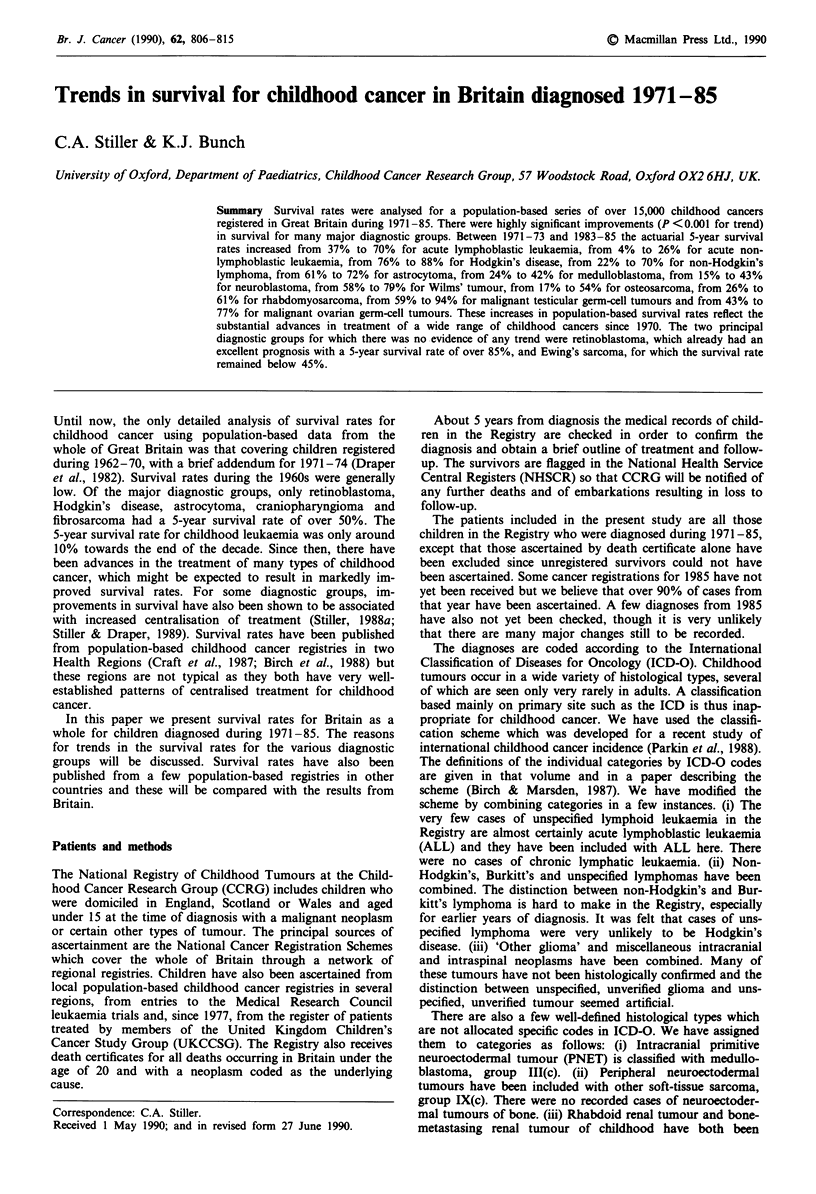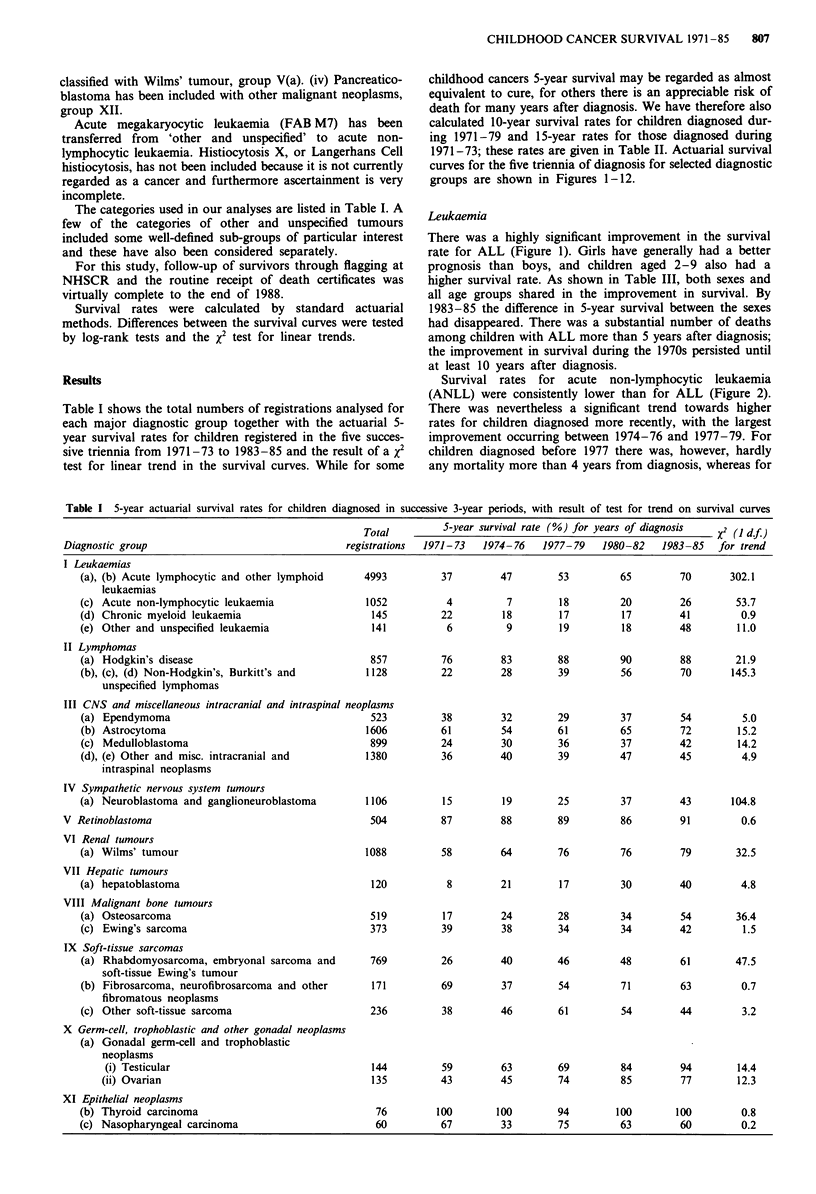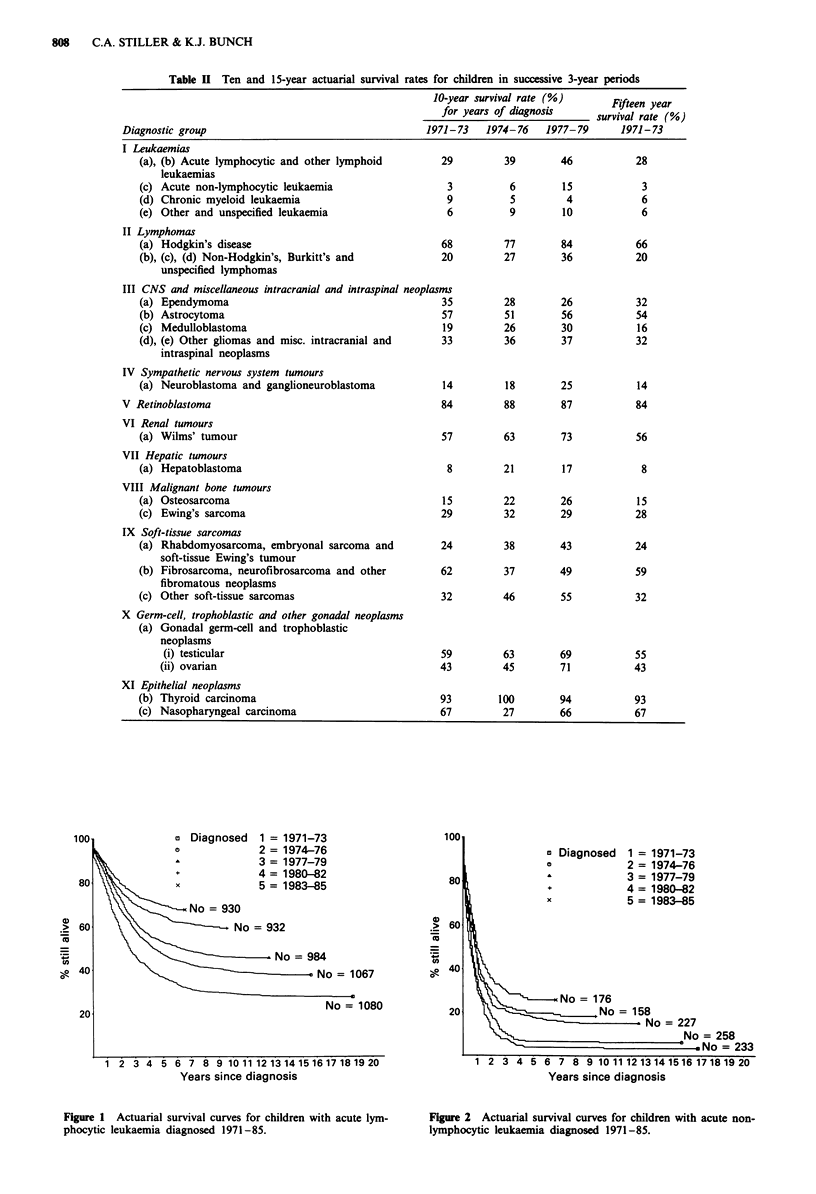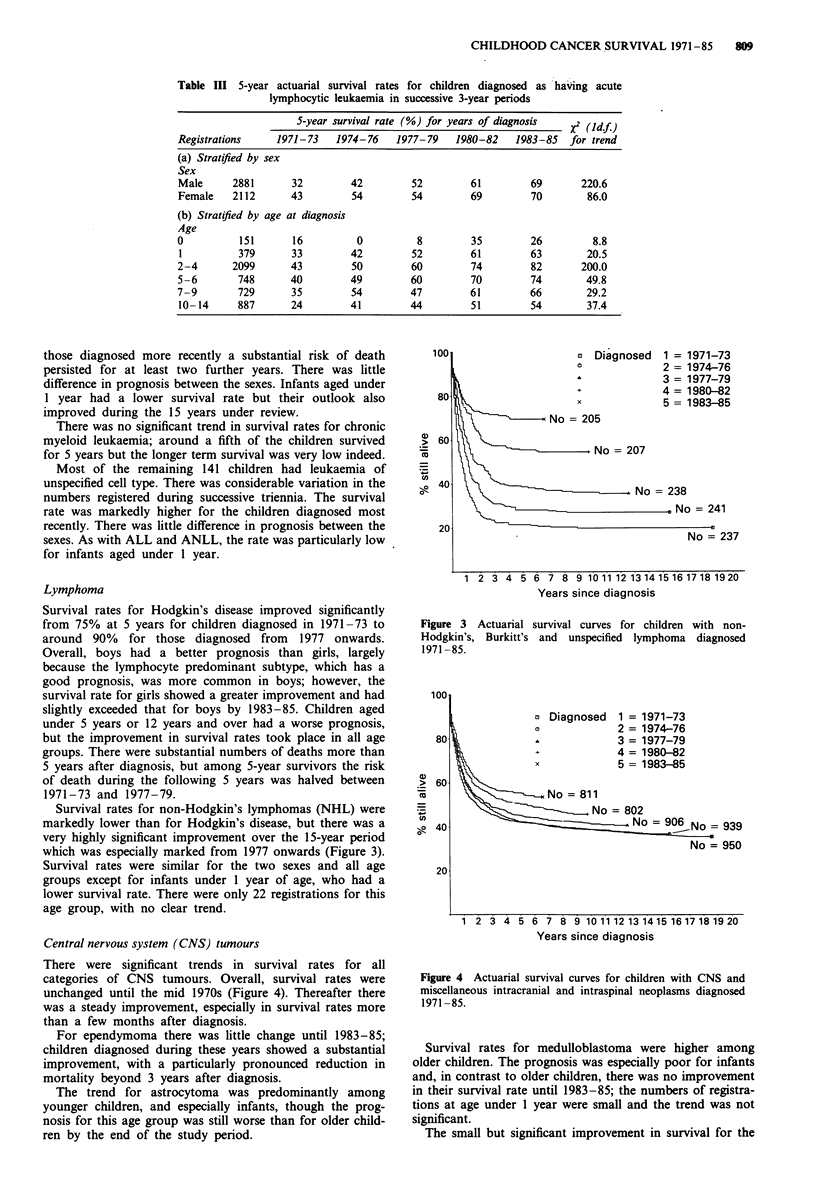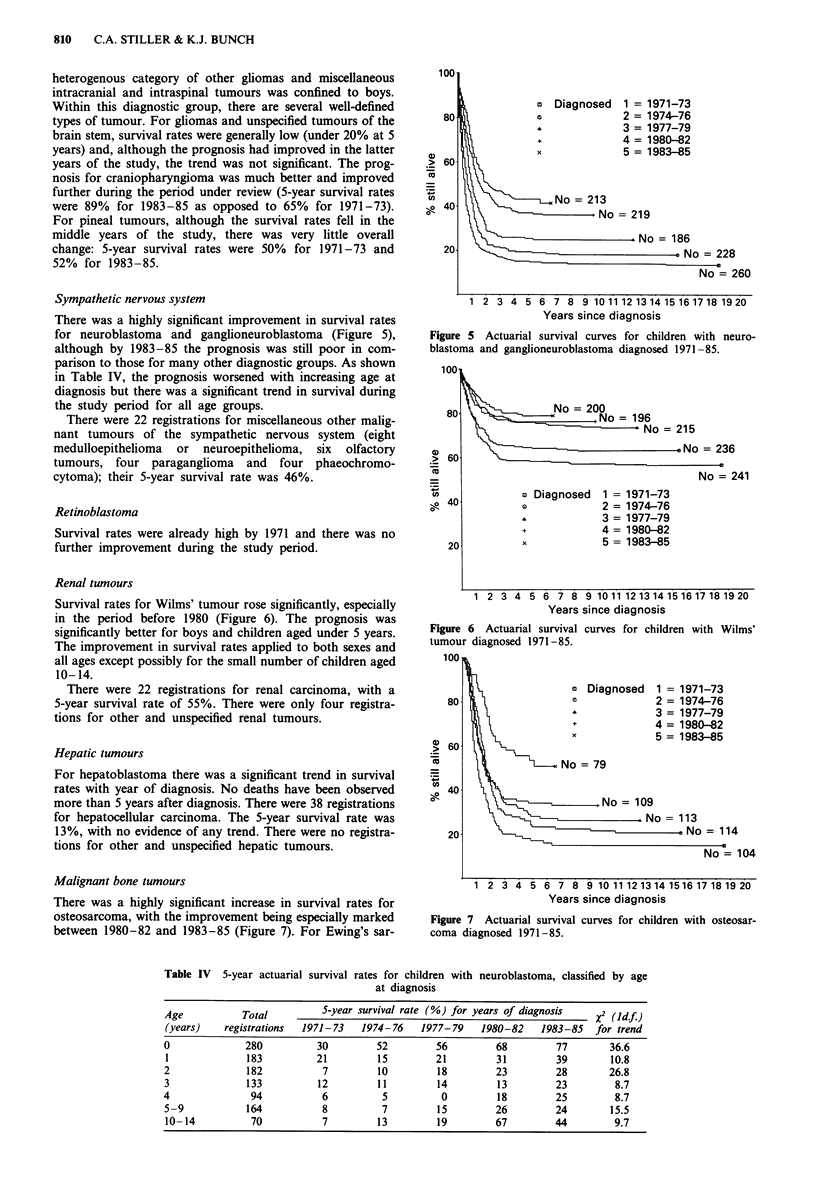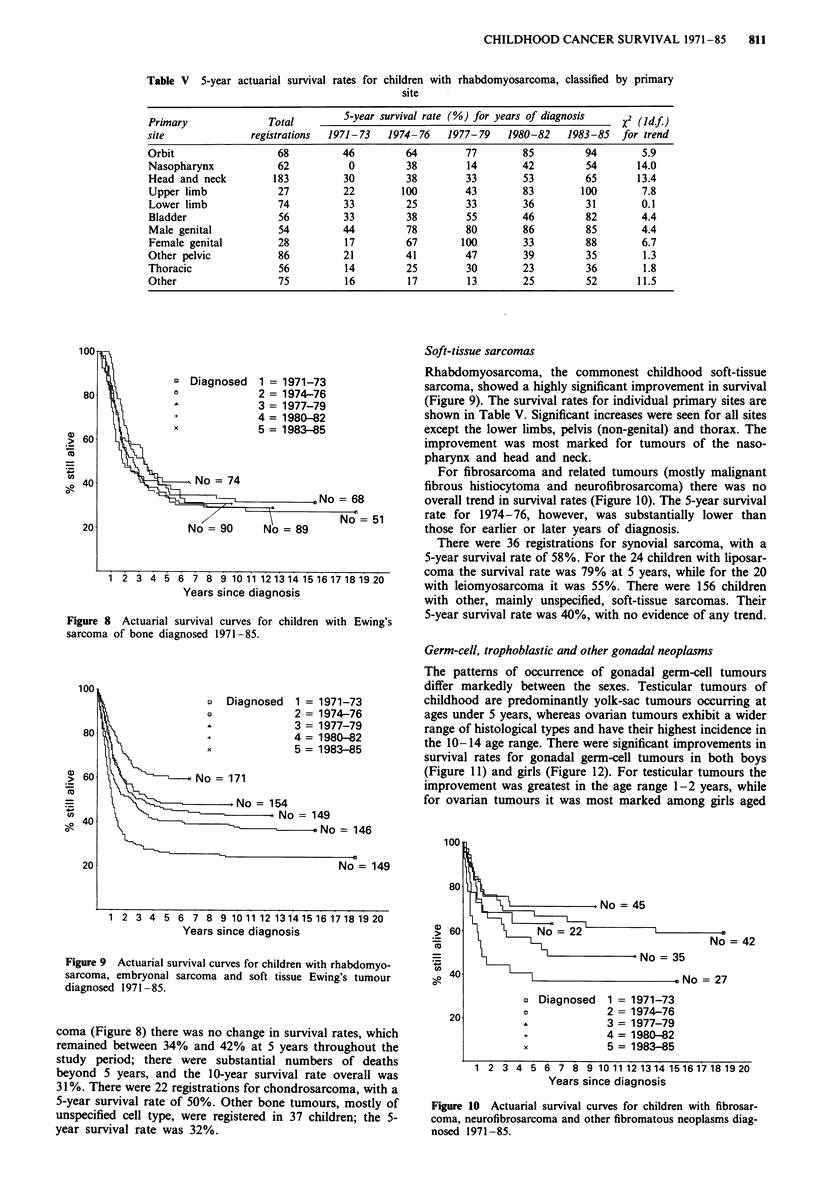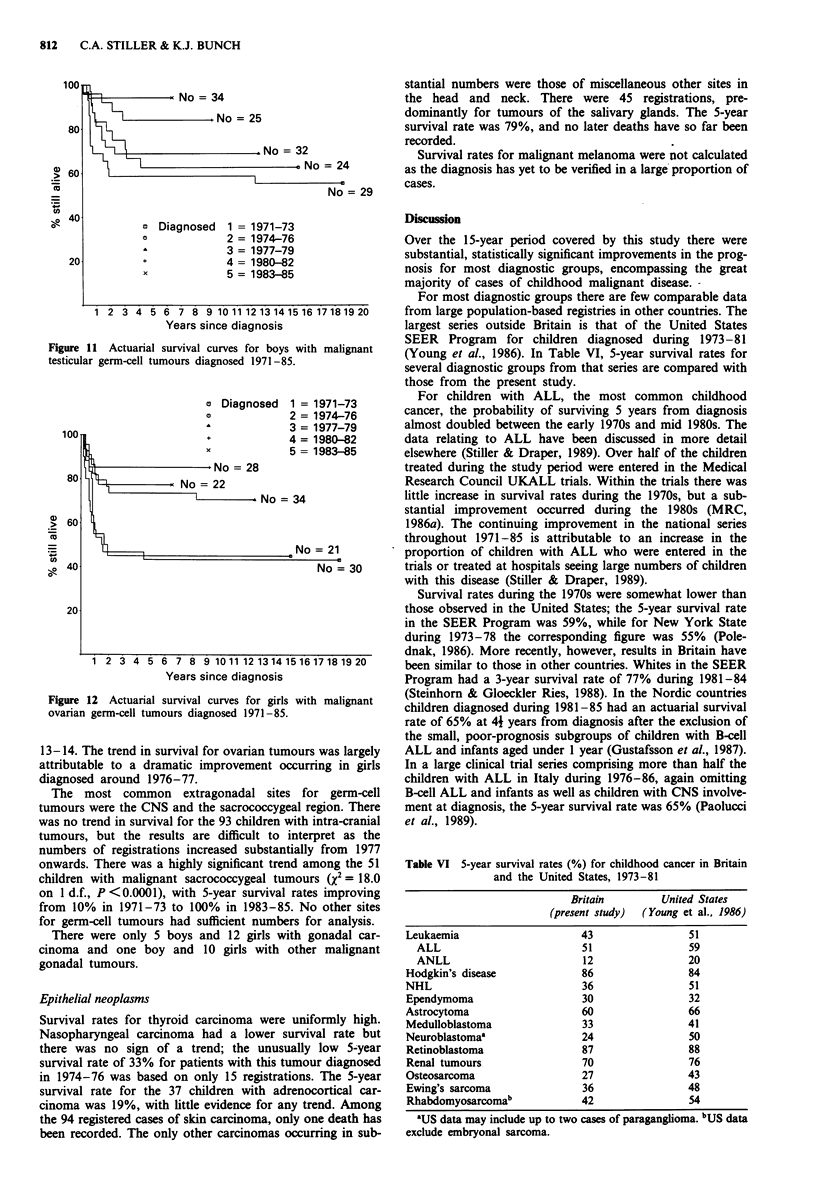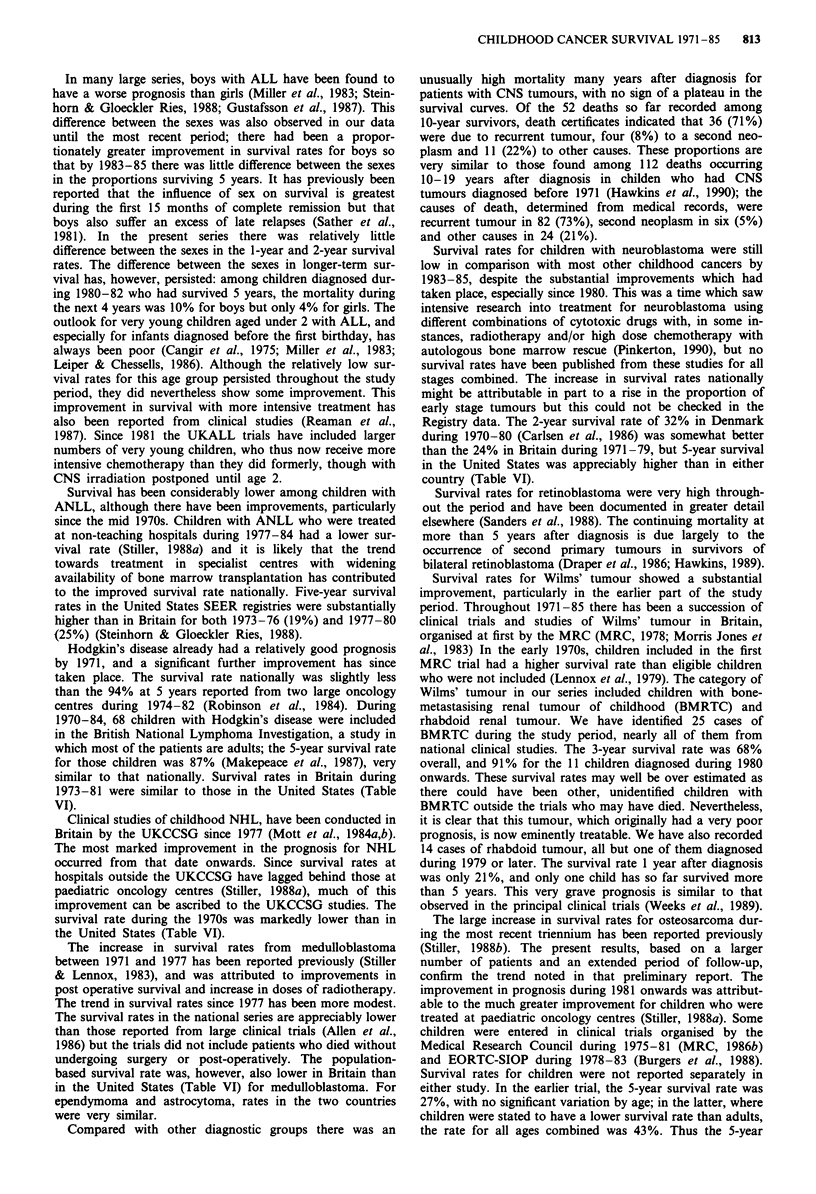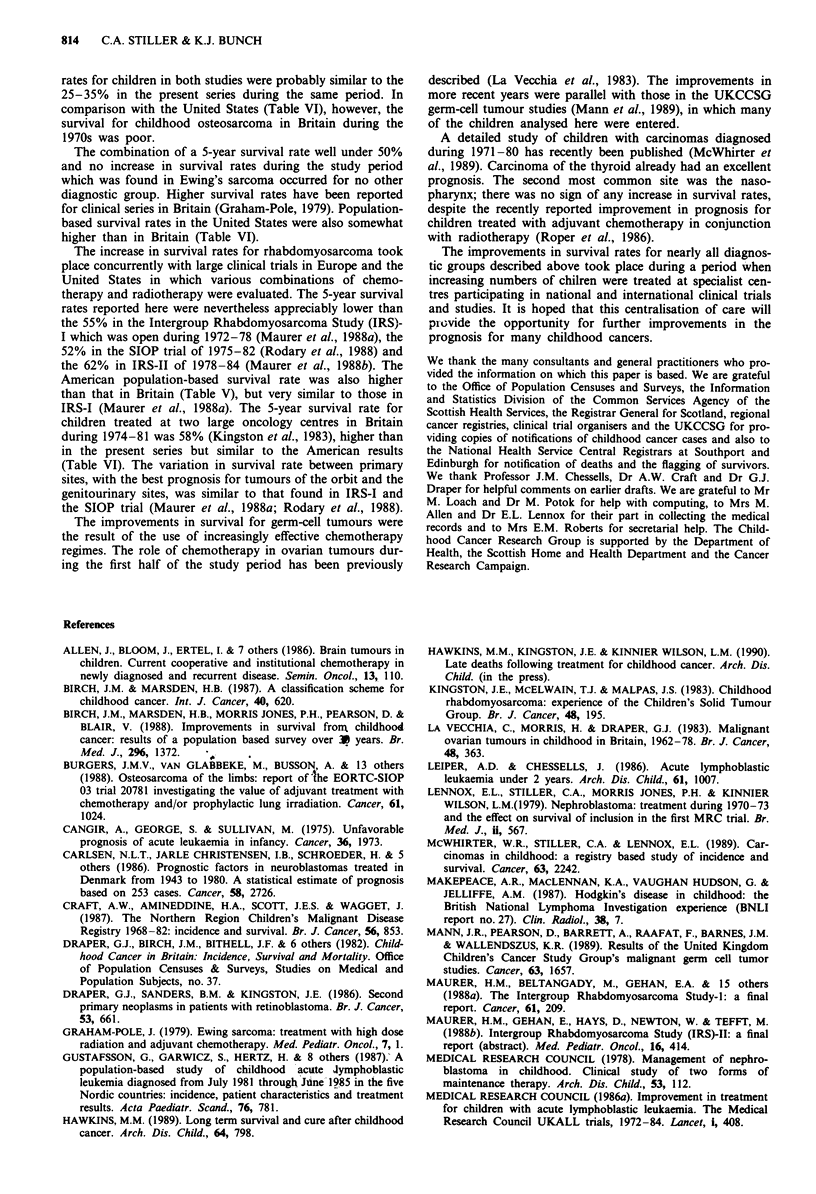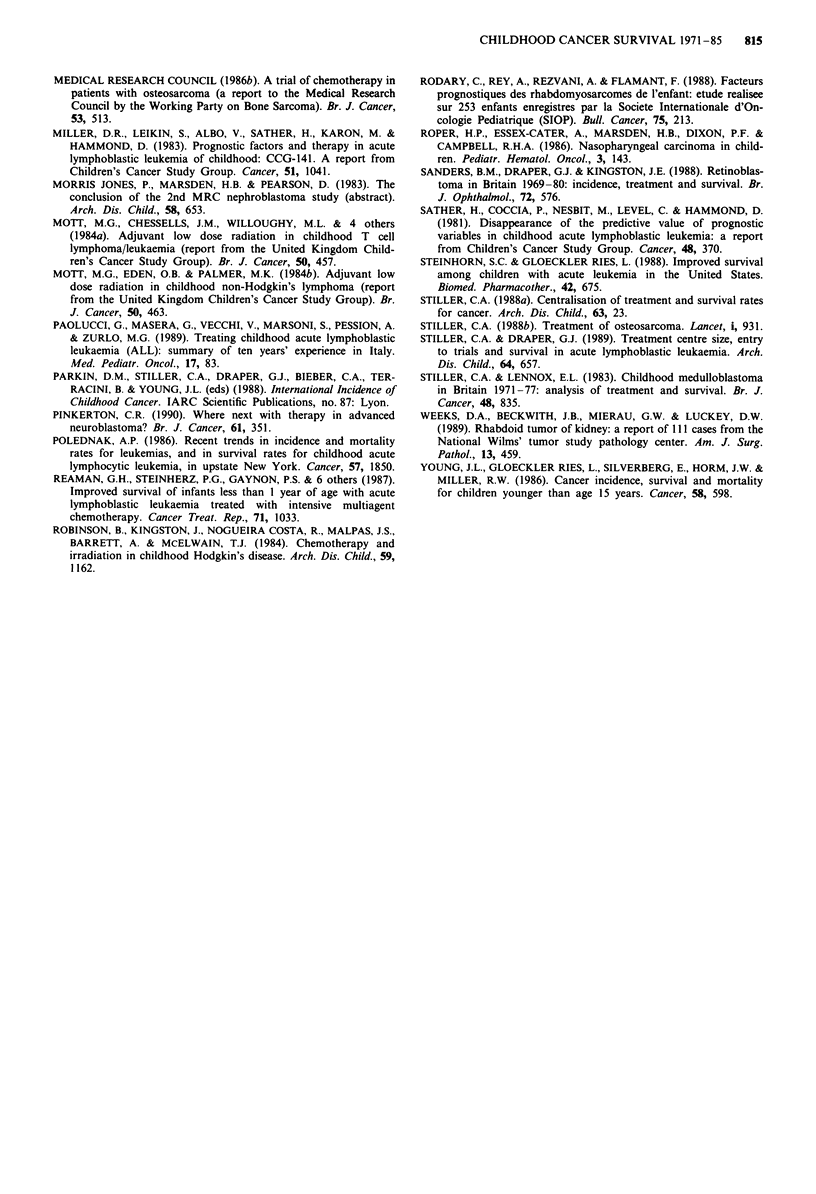# Trends in survival for childhood cancer in Britain diagnosed 1971-85.

**DOI:** 10.1038/bjc.1990.383

**Published:** 1990-11

**Authors:** C. A. Stiller, K. J. Bunch

**Affiliations:** University of Oxford, Department of Paediatrics, UK.

## Abstract

Survival rates were analysed for a population-based series of over 15,000 childhood cancers registered in Great Britain during 1971-85. There were highly significant improvements (P less than 0.001 for trend) in survival for many major diagnostic groups. Between 1971-73 and 1983-85 the actuarial 5-year survival rates increased from 37% to 70% for acute lymphoblastic leukaemia, from 4% to 26% for acute non-lymphoblastic leukaemia, from 76% to 88% for Hodgkin's disease, from 22% to 70% for non-Hodgkin's lymphoma, from 61% to 72% for astrocytoma, from 24% to 42% for medulloblastoma, from 15% to 43% for neuroblastoma, from 58% to 79% for Wilms' tumour, from 17% to 54% for osteosarcoma, from 26% to 61% for rhabdomyosarcoma, from 59% to 94% for malignant testicular germ-cell tumours and from 43% to 77% for malignant ovarian germ-cell tumours. These increases in population-based survival rates reflect the substantial advances in treatment of a wide range of childhood cancers since 1970. The two principal diagnostic groups for which there was no evidence of any trend were retinoblastoma, which already had an excellent prognosis with a 5-year survival rate of over 85%, and Ewing's sarcoma, for which the survival rate remained below 45%.


Br. J. Cancer (1990), 62, 806–815 © Macmillan Press Ltd., 1990

### Trends in survival for childhood cancer in Britain diagnosed 1971–85

C.A. Stiller & K.J. Bunch

*University of Oxford, Department of Paediatrics, Childhood Cancer Research Group, 57 Woodstock Road, Oxford OX2 6HJ, UK.*

**Summary** Survival rates were analysed for a population-based series of over 15,000 childhood cancers registered in Great Britain during 1971–85. There were highly significant improvements ($P < 0.001$ for trend) in survival for many major diagnostic groups. Between 1971–73 and 1983–85 the actuarial 5-year survival rates increased from 37% to 70% for acute lymphoblastic leukaemia, from 4% to 26% for acute non-lymphoblastic leukaemia, from 76% to 88% for Hodgkin's disease, from 22% to 70% for non-Hodgkin's lymphoma, from 61% to 72% for astrocytoma, from 24% to 42% for medulloblastoma, from 15% to 43% for neuroblastoma, from 58% to 79% for Wilms' tumour, from 17% to 54% for osteosarcoma, from 26% to 61% for rhabdomyosarcoma, from 59% to 94% for malignant testicular germ-cell tumours and from 43% to 77% for malignant ovarian germ-cell tumours. These increases in population-based survival rates reflect the substantial advances in treatment of a wide range of childhood cancers since 1970. The two principal diagnostic groups for which there was no evidence of any trend were retinoblastoma, which already had an excellent prognosis with a 5-year survival rate of over 85%, and Ewing's sarcoma, for which the survival rate remained below 45%.

Until now, the only detailed analysis of survival rates for childhood cancer using population-based data from the whole of Great Britain was that covering children registered during 1962–70, with a brief addendum for 1971–74 (Draper *et al.*, 1982). Survival rates during the 1960s were generally low. Of the major diagnostic groups, only retinoblastoma, Hodgkin's disease, astrocytoma, craniopharyngioma and fibrosarcoma had a 5-year survival rate of over 50%. The 5-year survival rate for childhood leukaemia was only around 10% towards the end of the decade. Since then, there have been advances in the treatment of many types of childhood cancer, which might be expected to result in markedly improved survival rates. For some diagnostic groups, improvements in survival have also been shown to be associated with increased centralisation of treatment (Stiller, 1988*a*; Stiller & Draper, 1989). Survival rates have been published from population-based childhood cancer registries in two Health Regions (Craft *et al.*, 1987; Birch *et al.*, 1988) but these regions are not typical as they both have very well-established patterns of centralised treatment for childhood cancer.

In this paper we present survival rates for Britain as a whole for children diagnosed during 1971–85. The reasons for trends in the survival rates for the various diagnostic groups will be discussed. Survival rates have also been published from a few population-based registries in other countries and these will be compared with the results from Britain.

### Patients and methods

The National Registry of Childhood Tumours at the Childhood Cancer Research Group (CCRG) includes children who were domiciled in England, Scotland or Wales and aged under 15 at the time of diagnosis with a malignant neoplasm or certain other types of tumour. The principal sources of ascertainment are the National Cancer Registration Schemes which cover the whole of Britain through a network of regional registries. Children have also been ascertained from local population-based childhood cancer registries in several regions, from entries to the Medical Research Council leukaemia trials and, since 1977, from the register of patients treated by members of the United Kingdom Children's Cancer Study Group (UKCCSG). The Registry also receives death certificates for all deaths occurring in Britain under the age of 20 and with a neoplasm coded as the underlying cause.

Correspondence: C.A. Stiller.
Received 1 May 1990; and in revised form 27 June 1990.

About 5 years from diagnosis the medical records of children in the Registry are checked in order to confirm the diagnosis and obtain a brief outline of treatment and follow-up. The survivors are flagged in the National Health Service Central Registers (NHSCR) so that CCRG will be notified of any further deaths and of embarkations resulting in loss to follow-up.

The patients included in the present study are all those children in the Registry who were diagnosed during 1971–85, except that those ascertained by death certificate alone have been excluded since unregistered survivors could not have been ascertained. Some cancer registrations for 1985 have not yet been received but we believe that over 90% of cases from that year have been ascertained. A few diagnoses from 1985 have also not yet been checked, though it is very unlikely that there are many major changes still to be recorded.

The diagnoses are coded according to the International Classification of Diseases for Oncology (ICD-O). Childhood tumours occur in a wide variety of histological types, several of which are seen only very rarely in adults. A classification based mainly on primary site such as the ICD is thus inappropriate for childhood cancer. We have used the classification scheme which was developed for a recent study of international childhood cancer incidence (Parkin *et al.*, 1988). The definitions of the individual categories by ICD-O codes are given in that volume and in a paper describing the scheme (Birch & Marsden, 1987). We have modified the scheme by combining categories in a few instances. (i) The very few cases of unspecified lymphoid leukaemia in the Registry are almost certainly acute lymphoblastic leukaemia (ALL) and they have been included with ALL here. There were no cases of chronic lymphatic leukaemia. (ii) Non-Hodgkin's, Burkitt's and unspecified lymphomas have been combined. The distinction between non-Hodgkin's and Burkitt's lymphoma is hard to make in the Registry, especially for earlier years of diagnosis. It was felt that cases of unspecified lymphoma were very unlikely to be Hodgkin's disease. (iii) 'Other glioma' and miscellaneous intracranial and intraspinal neoplasms have been combined. Many of these tumours have not been histologically confirmed and the distinction between unspecified, unverified glioma and unspecified, unverified tumour seemed artificial.

There are also a few well-defined histological types which are not allocated specific codes in ICD-O. We have assigned them to categories as follows: (i) Intracranial primitive neuroectodermal tumour (PNET) is classified with medulloblastoma, group III(c). (ii) Peripheral neuroectodermal tumours have been included with other soft-tissue sarcoma, group IX(c). There were no recorded cases of neuroectodermal tumours of bone. (iii) Rhabdoid renal tumour and bone-metastasing renal tumour of childhood have both been classified with Wilms' tumour, group V(a). (iv) Pancreaticoblastoma has been included with other malignant neoplasms, group XII.

Acute megakaryocytic leukaemia (FAB M7) has been transferred from 'other and unspecified' to acute non-lymphocytic leukaemia. Histiocytosis X, or Langerhans Cell histiocytosis, has not been included because it is not currently regarded as a cancer and furthermore ascertainment is very incomplete.

The categories used in our analyses are listed in Table I. A few of the categories of other and unspecified tumours included some well-defined sub-groups of particular interest and these have also been considered separately.

For this study, follow-up of survivors through flagging at NHSCR and the routine receipt of death certificates was virtually complete to the end of 1988.

Survival rates were calculated by standard actuarial methods. Differences between the survival curves were tested by log-rank tests and the $\chi^2$ test for linear trends.

### Results

Table I shows the total numbers of registrations analysed for each major diagnostic group together with the actuarial 5-year survival rates for children registered in the five successive triennia from 1971-73 to 1983-85 and the result of a $\chi^2$ test for linear trend in the survival curves. While for some childhood cancers 5-year survival may be regarded as almost equivalent to cure, for others there is an appreciable risk of death for many years after diagnosis. We have therefore also calculated 10-year survival rates for children diagnosed during 1971-79 and 15-year rates for those diagnosed during 1971-73; these rates are given in Table II. Actuarial survival curves for the five triennia of diagnosis for selected diagnostic groups are shown in Figures 1-12.

### *Leukaemia*

There was a highly significant improvement in the survival rate for ALL (Figure 1). Girls have generally had a better prognosis than boys, and children aged 2-9 also had a higher survival rate. As shown in Table III, both sexes and all age groups shared in the improvement in survival. By 1983-85 the difference in 5-year survival between the sexes had disappeared. There was a substantial number of deaths among children with ALL more than 5 years after diagnosis; the improvement in survival during the 1970s persisted until at least 10 years after diagnosis.

Survival rates for acute non-lymphocytic leukaemia (ANLL) were consistently lower than for ALL (Figure 2). There was nevertheless a significant trend towards higher rates for children diagnosed more recently, with the largest improvement occurring between 1974-76 and 1977-79. For children diagnosed before 1977 there was, however, hardly any mortality more than 4 years from diagnosis, whereas for

**Table I** 5-year actuarial survival rates for children diagnosed in successive 3-year periods, with result of test for trend on survival curves

| Diagnostic group | Total registrations | *5-year survival rate (%) for years of diagnosis* 1971-73 | 1974-76 | 1977-79 | 1980-82 | 1983-85 | $\chi^2$ *(1 d.f.) for trend* |
|---|---|---|---|---|---|---|---|
| I *Leukaemias* | | | | | | | |
| (a), (b) Acute lymphocytic and other lymphoid leukaemias | 4993 | 37 | 47 | 53 | 65 | 70 | 302.1 |
| (c) Acute non-lymphocytic leukaemia | 1052 | 4 | 7 | 18 | 20 | 26 | 53.7 |
| (d) Chronic myeloid leukaemia | 145 | 22 | 18 | 17 | 17 | 41 | 0.9 |
| (e) Other and unspecified leukaemia | 141 | 6 | 9 | 19 | 18 | 48 | 11.0 |
| II *Lymphomas* | | | | | | | |
| (a) Hodgkin's disease | 857 | 76 | 83 | 88 | 90 | 88 | 21.9 |
| (b), (c), (d) Non-Hodgkin's, Burkitt's and unspecified lymphomas | 1128 | 22 | 28 | 39 | 56 | 70 | 145.3 |
| III *CNS and miscellaneous intracranial and intraspinal neoplasms* | | | | | | | |
| (a) Ependymoma | 523 | 38 | 32 | 29 | 37 | 54 | 5.0 |
| (b) Astrocytoma | 1606 | 61 | 54 | 61 | 65 | 72 | 15.2 |
| (c) Medulloblastoma | 899 | 24 | 30 | 36 | 37 | 42 | 14.2 |
| (d), (e) Other and misc. intracranial and intraspinal neoplasms | 1380 | 36 | 40 | 39 | 47 | 45 | 4.9 |
| IV *Sympathetic nervous system tumours* | | | | | | | |
| (a) Neuroblastoma and ganglioneuroblastoma | 1106 | 15 | 19 | 25 | 37 | 43 | 104.8 |
| V *Retinoblastoma* | 504 | 87 | 88 | 89 | 86 | 91 | 0.6 |
| VI *Renal tumours* | | | | | | | |
| (a) Wilms' tumour | 1088 | 58 | 64 | 76 | 76 | 79 | 32.5 |
| VII *Hepatic tumours* | | | | | | | |
| (a) hepatoblastoma | 120 | 8 | 21 | 17 | 30 | 40 | 4.8 |
| VIII *Malignant bone tumours* | | | | | | | |
| (a) Osteosarcoma | 519 | 17 | 24 | 28 | 34 | 54 | 36.4 |
| (c) Ewing's sarcoma | 373 | 39 | 38 | 34 | 34 | 42 | 1.5 |
| IX *Soft-tissue sarcomas* | | | | | | | |
| (a) Rhabdomyosarcoma, embryonal sarcoma and soft-tissue Ewing's tumour | 769 | 26 | 40 | 46 | 48 | 61 | 47.5 |
| (b) Fibrosarcoma, neurofibrosarcoma and other fibromatous neoplasms | 171 | 69 | 37 | 54 | 71 | 63 | 0.7 |
| (c) Other soft-tissue sarcoma | 236 | 38 | 46 | 61 | 54 | 44 | 3.2 |
| X *Germ-cell, trophoblastic and other gonadal neoplasms* | | | | | | | |
| (a) Gonadal germ-cell and trophoblastic neoplasms | | | | | | | |
| (i) Testicular | 144 | 59 | 63 | 69 | 84 | 94 | 14.4 |
| (ii) Ovarian | 135 | 43 | 45 | 74 | 85 | 77 | 12.3 |
| XI *Epithelial neoplasms* | | | | | | | |
| (b) Thyroid carcinoma | 76 | 100 | 100 | 94 | 100 | 100 | 0.8 |
| (c) Nasopharyngeal carcinoma | 60 | 67 | 33 | 75 | 63 | 60 | 0.2 |

**Table II** Ten and 15-year actuarial survival rates for children in successive 3-year periods

| | *10-year survival rate (%) for years of diagnosis* | | | *Fifteen year survival rate (%)* |
|---|---|---|---|---|
| *Diagnostic group* | *1971–73* | *1974–76* | *1977–79* | *1971–73* |
| I *Leukaemias* | | | | |
| (a), (b) Acute lymphocytic and other lymphoid leukaemias | 29 | 39 | 46 | 28 |
| (c) Acute non-lymphocytic leukaemia | 3 | 6 | 15 | 3 |
| (d) Chronic myeloid leukaemia | 9 | 5 | 4 | 6 |
| (e) Other and unspecified leukaemia | 6 | 9 | 10 | 6 |
| II *Lymphomas* | | | | |
| (a) Hodgkin's disease | 68 | 77 | 84 | 66 |
| (b), (c), (d) Non-Hodgkin's, Burkitt's and unspecified lymphomas | 20 | 27 | 36 | 20 |
| III *CNS and miscellaneous intracranial and intraspinal neoplasms* | | | | |
| (a) Ependymoma | 35 | 28 | 26 | 32 |
| (b) Astrocytoma | 57 | 51 | 56 | 54 |
| (c) Medulloblastoma | 19 | 26 | 30 | 16 |
| (d), (e) Other gliomas and misc. intracranial and intraspinal neoplasms | 33 | 36 | 37 | 32 |
| IV *Sympathetic nervous system tumours* | | | | |
| (a) Neuroblastoma and ganglioneuroblastoma | 14 | 18 | 25 | 14 |
| V *Retinoblastoma* | 84 | 88 | 87 | 84 |
| VI *Renal tumours* | | | | |
| (a) Wilms' tumour | 57 | 63 | 73 | 56 |
| VII *Hepatic tumours* | | | | |
| (a) Hepatoblastoma | 8 | 21 | 17 | 8 |
| VIII *Malignant bone tumours* | | | | |
| (a) Osteosarcoma | 15 | 22 | 26 | 15 |
| (c) Ewing's sarcoma | 29 | 32 | 29 | 28 |
| IX *Soft-tissue sarcomas* | | | | |
| (a) Rhabdomyosarcoma, embryonal sarcoma and soft-tissue Ewing's tumour | 24 | 38 | 43 | 24 |
| (b) Fibrosarcoma, neurofibrosarcoma and other fibromatous neoplasms | 62 | 37 | 49 | 59 |
| (c) Other soft-tissue sarcomas | 32 | 46 | 55 | 32 |
| X *Germ-cell, trophoblastic and other gonadal neoplasms* | | | | |
| (a) Gonadal germ-cell and trophoblastic neoplasms | | | | |
| (i) testicular | 59 | 63 | 69 | 55 |
| (ii) ovarian | 43 | 45 | 71 | 43 |
| XI *Epithelial neoplasms* | | | | |
| (b) Thyroid carcinoma | 93 | 100 | 94 | 93 |
| (c) Nasopharyngeal carcinoma | 67 | 27 | 66 | 67 |

**Figure 1** Actuarial survival curves for children with acute lymphocytic leukaemia diagnosed 1971–85.

**Figure 2** Actuarial survival curves for children with acute non-lymphocytic leukaemia diagnosed 1971–85.

**Table III** 5-year actuarial survival rates for children diagnosed as having acute lymphocytic leukaemia in successive 3-year periods

| Registrations | | 5-year survival rate (%) for years of diagnosis 1971-73 | 1974-76 | 1977-79 | 1980-82 | 1983-85 | $\chi^2$ (1d.f.) for trend |
|---|---|---|---|---|---|---|---|
| (a) *Stratified by sex* | | | | | | | |
| *Sex* | | | | | | | |
| Male | 2881 | 32 | 42 | 52 | 61 | 69 | 220.6 |
| Female | 2112 | 43 | 54 | 54 | 69 | 70 | 86.0 |
| (b) *Stratified by age at diagnosis* | | | | | | | |
| *Age* | | | | | | | |
| 0 | 151 | 16 | 0 | 8 | 35 | 26 | 8.8 |
| 1 | 379 | 33 | 42 | 52 | 61 | 63 | 20.5 |
| 2-4 | 2099 | 43 | 50 | 60 | 74 | 82 | 200.0 |
| 5-6 | 748 | 40 | 49 | 60 | 70 | 74 | 49.8 |
| 7-9 | 729 | 35 | 54 | 47 | 61 | 66 | 29.2 |
| 10-14 | 887 | 24 | 41 | 44 | 51 | 54 | 37.4 |

those diagnosed more recently a substantial risk of death persisted for at least two further years. There was little difference in prognosis between the sexes. Infants aged under 1 year had a lower survival rate but their outlook also improved during the 15 years under review.

There was no significant trend in survival rates for chronic myeloid leukaemia; around a fifth of the children survived for 5 years but the longer term survival was very low indeed.

Most of the remaining 141 children had leukaemia of unspecified cell type. There was considerable variation in the numbers registered during successive triennia. The survival rate was markedly higher for the children diagnosed most recently. There was little difference in prognosis between the sexes. As with ALL and ANLL, the rate was particularly low for infants aged under 1 year.

### *Lymphoma*

Survival rates for Hodgkin's disease improved significantly from 75% at 5 years for children diagnosed in 1971-73 to around 90% for those diagnosed from 1977 onwards. Overall, boys had a better prognosis than girls, largely because the lymphocyte predominant subtype, which has a good prognosis, was more common in boys; however, the survival rate for girls showed a greater improvement and had slightly exceeded that for boys by 1983-85. Children aged under 5 years or 12 years and over had a worse prognosis, but the improvement in survival rates took place in all age groups. There were substantial numbers of deaths more than 5 years after diagnosis, but among 5-year survivors the risk of death during the following 5 years was halved between 1971-73 and 1977-79.

Survival rates for non-Hodgkin's lymphomas (NHL) were markedly lower than for Hodgkin's disease, but there was a very highly significant improvement over the 15-year period which was especially marked from 1977 onwards (Figure 3). Survival rates were similar for the two sexes and all age groups except for infants under 1 year of age, who had a lower survival rate. There were only 22 registrations for this age group, with no clear trend.

### *Central nervous system (CNS) tumours*

There were significant trends in survival rates for all categories of CNS tumours. Overall, survival rates were unchanged until the mid 1970s (Figure 4). Thereafter there was a steady improvement, especially in survival rates more than a few months after diagnosis.

For ependymoma there was little change until 1983-85; children diagnosed during these years showed a substantial improvement, with a particularly pronounced reduction in mortality beyond 3 years after diagnosis.

The trend for astrocytoma was predominantly among younger children, and especially infants, though the prognosis for this age group was still worse than for older children by the end of the study period.

**Figure 3** Actuarial survival curves for children with non-Hodgkin's, Burkitt's and unspecified lymphoma diagnosed 1971-85.

**Figure 4** Actuarial survival curves for children with CNS and miscellaneous intracranial and intraspinal neoplasms diagnosed 1971-85.

Survival rates for medulloblastoma were higher among older children. The prognosis was especially poor for infants and, in contrast to older children, there was no improvement in their survival rate until 1983-85; the numbers of registrations at age under 1 year were small and the trend was not significant.

The small but significant improvement in survival for the heterogenous category of other gliomas and miscellaneous intracranial and intraspinal tumours was confined to boys. Within this diagnostic group, there are several well-defined types of tumour. For gliomas and unspecified tumours of the brain stem, survival rates were generally low (under 20% at 5 years) and, although the prognosis had improved in the latter years of the study, the trend was not significant. The prognosis for craniopharyngioma was much better and improved further during the period under review (5-year survival rates were 89% for 1983-85 as opposed to 65% for 1971-73). For pineal tumours, although the survival rates fell in the middle years of the study, there was very little overall change: 5-year survival rates were 50% for 1971-73 and 52% for 1983-85.

### *Sympathetic nervous system*

There was a highly significant improvement in survival rates for neuroblastoma and ganglioneuroblastoma (Figure 5), although by 1983-85 the prognosis was still poor in comparison to those for many other diagnostic groups. As shown in Table IV, the prognosis worsened with increasing age at diagnosis but there was a significant trend in survival during the study period for all age groups.

There were 22 registrations for miscellaneous other malignant tumours of the sympathetic nervous system (eight medulloepithelioma or neuroepithelioma, six olfactory tumours, four paraganglioma and four phaeochromocytoma); their 5-year survival rate was 46%.

### *Retinoblastoma*

Survival rates were already high by 1971 and there was no further improvement during the study period.

### *Renal tumours*

Survival rates for Wilms' tumour rose significantly, especially in the period before 1980 (Figure 6). The prognosis was significantly better for boys and children aged under 5 years. The improvement in survival rates applied to both sexes and all ages except possibly for the small number of children aged 10-14.

There were 22 registrations for renal carcinoma, with a 5-year survival rate of 55%. There were only four registrations for other and unspecified renal tumours.

### *Hepatic tumours*

For hepatoblastoma there was a significant trend in survival rates with year of diagnosis. No deaths have been observed more than 5 years after diagnosis. There were 38 registrations for hepatocellular carcinoma. The 5-year survival rate was 13%, with no evidence of any trend. There were no registrations for other and unspecified hepatic tumours.

### *Malignant bone tumours*

There was a highly significant increase in survival rates for osteosarcoma, with the improvement being especially marked between 1980-82 and 1983-85 (Figure 7). For Ewing's sar-

**Figure 5** Actuarial survival curves for children with neuroblastoma and ganglioneuroblastoma diagnosed 1971-85.

**Figure 6** Actuarial survival curves for children with Wilms' tumour diagnosed 1971-85.

**Figure 7** Actuarial survival curves for children with osteosarcoma diagnosed 1971-85.

**Table IV** 5-year actuarial survival rates for children with neuroblastoma, classified by age at diagnosis

| *Age (years)* | *Total registrations* | *5-year survival rate (%) for years of diagnosis* 1971-73 | 1974-76 | 1977-79 | 1980-82 | 1983-85 | $\chi^2$ *(1d.f.) for trend* |
|---|---|---|---|---|---|---|---|
| 0 | 280 | 30 | 52 | 56 | 68 | 77 | 36.6 |
| 1 | 183 | 21 | 15 | 21 | 31 | 39 | 10.8 |
| 2 | 182 | 7 | 10 | 18 | 23 | 28 | 26.8 |
| 3 | 133 | 12 | 11 | 14 | 13 | 23 | 8.7 |
| 4 | 94 | 6 | 5 | 0 | 18 | 25 | 8.7 |
| 5-9 | 164 | 8 | 7 | 15 | 26 | 24 | 15.5 |
| 10-14 | 70 | 7 | 13 | 19 | 67 | 44 | 9.7 |

**Table V** 5-year actuarial survival rates for children with rhabdomyosarcoma, classified by primary site

| *Primary site* | *Total registrations* | *5-year survival rate (%) for years of diagnosis* 1971-73 | 1974-76 | 1977-79 | 1980-82 | 1983-85 | $\chi^2$ *(1d.f.) for trend* |
|---|---|---|---|---|---|---|---|
| Orbit | 68 | 46 | 64 | 77 | 85 | 94 | 5.9 |
| Nasopharynx | 62 | 0 | 38 | 14 | 42 | 54 | 14.0 |
| Head and neck | 183 | 30 | 38 | 33 | 53 | 65 | 13.4 |
| Upper limb | 27 | 22 | 100 | 43 | 83 | 100 | 7.8 |
| Lower limb | 74 | 33 | 25 | 33 | 36 | 31 | 0.1 |
| Bladder | 56 | 33 | 38 | 55 | 46 | 82 | 4.4 |
| Male genital | 54 | 44 | 78 | 80 | 86 | 85 | 4.4 |
| Female genital | 28 | 17 | 67 | 100 | 33 | 88 | 6.7 |
| Other pelvic | 86 | 21 | 41 | 47 | 39 | 35 | 1.3 |
| Thoracic | 56 | 14 | 25 | 30 | 23 | 36 | 1.8 |
| Other | 75 | 16 | 17 | 13 | 25 | 52 | 11.5 |

**Figure 8** Actuarial survival curves for children with Ewing's sarcoma of bone diagnosed 1971-85.

**Figure 9** Actuarial survival curves for children with rhabdomyosarcoma, embryonal sarcoma and soft tissue Ewing's tumour diagnosed 1971-85.

coma (Figure 8) there was no change in survival rates, which remained between 34% and 42% at 5 years throughout the study period; there were substantial numbers of deaths beyond 5 years, and the 10-year survival rate overall was 31%. There were 22 registrations for chondrosarcoma, with a 5-year survival rate of 50%. Other bone tumours, mostly of unspecified cell type, were registered in 37 children; the 5-year survival rate was 32%.

*Soft-tissue sarcomas*

Rhabdomyosarcoma, the commonest childhood soft-tissue sarcoma, showed a highly significant improvement in survival (Figure 9). The survival rates for individual primary sites are shown in Table V. Significant increases were seen for all sites except the lower limbs, pelvis (non-genital) and thorax. The improvement was most marked for tumours of the nasopharynx and head and neck.

For fibrosarcoma and related tumours (mostly malignant fibrous histiocytoma and neurofibrosarcoma) there was no overall trend in survival rates (Figure 10). The 5-year survival rate for 1974-76, however, was substantially lower than those for earlier or later years of diagnosis.

There were 36 registrations for synovial sarcoma, with a 5-year survival rate of 58%. For the 24 children with liposarcoma the survival rate was 79% at 5 years, while for the 20 with leiomyosarcoma it was 55%. There were 156 children with other, mainly unspecified, soft-tissue sarcomas. Their 5-year survival rate was 40%, with no evidence of any trend.

*Germ-cell, trophoblastic and other gonadal neoplasms*

The patterns of occurrence of gonadal germ-cell tumours differ markedly between the sexes. Testicular tumours of childhood are predominantly yolk-sac tumours occurring at ages under 5 years, whereas ovarian tumours exhibit a wider range of histological types and have their highest incidence in the 10-14 age range. There were significant improvements in survival rates for gonadal germ-cell tumours in both boys (Figure 11) and girls (Figure 12). For testicular tumours the improvement was greatest in the age range 1-2 years, while for ovarian tumours it was most marked among girls aged

**Figure 10** Actuarial survival curves for children with fibrosarcoma, neurofibrosarcoma and other fibromatous neoplasms diagnosed 1971-85.

**Figure 11** Actuarial survival curves for boys with malignant testicular germ-cell tumours diagnosed 1971–85.

**Figure 12** Actuarial survival curves for girls with malignant ovarian germ-cell tumours diagnosed 1971–85.

13–14. The trend in survival for ovarian tumours was largely attributable to a dramatic improvement occurring in girls diagnosed around 1976–77.

The most common extragonadal sites for germ-cell tumours were the CNS and the sacrococcygeal region. There was no trend in survival for the 93 children with intra-cranial tumours, but the results are difficult to interpret as the numbers of registrations increased substantially from 1977 onwards. There was a highly significant trend among the 51 children with malignant sacrococcygeal tumours ($\chi^2 = 18.0$ on 1 d.f., $P < 0.0001$), with 5-year survival rates improving from 10% in 1971–73 to 100% in 1983–85. No other sites for germ-cell tumours had sufficient numbers for analysis.

There were only 5 boys and 12 girls with gonadal carcinoma and one boy and 10 girls with other malignant gonadal tumours.

### *Epithelial neoplasms*

Survival rates for thyroid carcinoma were uniformly high. Nasopharyngeal carcinoma had a lower survival rate but there was no sign of a trend; the unusually low 5-year survival rate of 33% for patients with this tumour diagnosed in 1974–76 was based on only 15 registrations. The 5-year survival rate for the 37 children with adrenocortical carcinoma was 19%, with little evidence for any trend. Among the 94 registered cases of skin carcinoma, only one death has been recorded. The only other carcinomas occurring in substantial numbers were those of miscellaneous other sites in the head and neck. There were 45 registrations, predominantly for tumours of the salivary glands. The 5-year survival rate was 79%, and no later deaths have so far been recorded.

Survival rates for malignant melanoma were not calculated as the diagnosis has yet to be verified in a large proportion of cases.

### Discussion

Over the 15-year period covered by this study there were substantial, statistically significant improvements in the prognosis for most diagnostic groups, encompassing the great majority of cases of childhood malignant disease.

For most diagnostic groups there are few comparable data from large population-based registries in other countries. The largest series outside Britain is that of the United States SEER Program for children diagnosed during 1973–81 (Young *et al.*, 1986). In Table VI, 5-year survival rates for several diagnostic groups from that series are compared with those from the present study.

For children with ALL, the most common childhood cancer, the probability of surviving 5 years from diagnosis almost doubled between the early 1970s and mid 1980s. The data relating to ALL have been discussed in more detail elsewhere (Stiller & Draper, 1989). Over half of the children treated during the study period were entered in the Medical Research Council UKALL trials. Within the trials there was little increase in survival rates during the 1970s, but a substantial improvement occurred during the 1980s (MRC, 1986*a*). The continuing improvement in the national series throughout 1971–85 is attributable to an increase in the proportion of children with ALL who were entered in the trials or treated at hospitals seeing large numbers of children with this disease (Stiller & Draper, 1989).

Survival rates during the 1970s were somewhat lower than those observed in the United States; the 5-year survival rate in the SEER Program was 59%, while for New York State during 1973–78 the corresponding figure was 55% (Polednak, 1986). More recently, however, results in Britain have been similar to those in other countries. Whites in the SEER Program had a 3-year survival rate of 77% during 1981–84 (Steinhorn & Gloeckler Ries, 1988). In the Nordic countries children diagnosed during 1981–85 had an actuarial survival rate of 65% at 4½ years from diagnosis after the exclusion of the small, poor-prognosis subgroups of children with B-cell ALL and infants aged under 1 year (Gustafsson *et al.*, 1987). In a large clinical trial series comprising more than half the children with ALL in Italy during 1976–86, again omitting B-cell ALL and infants as well as children with CNS involvement at diagnosis, the 5-year survival rate was 65% (Paolucci *et al.*, 1989).

**Table VI** 5-year survival rates (%) for childhood cancer in Britain and the United States, 1973–81

| | *Britain (present study)* | *United States (Young* et al., *1986)* |
|---|---|---|
| Leukaemia | 43 | 51 |
| ALL | 51 | 59 |
| ANLL | 12 | 20 |
| Hodgkin's disease | 86 | 84 |
| NHL | 36 | 51 |
| Ependymoma | 30 | 32 |
| Astrocytoma | 60 | 66 |
| Medulloblastoma | 33 | 41 |
| Neuroblastoma[a] | 24 | 50 |
| Retinoblastoma | 87 | 88 |
| Renal tumours | 70 | 76 |
| Osteosarcoma | 27 | 43 |
| Ewing's sarcoma | 36 | 48 |
| Rhabdomyosarcoma[b] | 42 | 54 |

[a]US data may include up to two cases of paraganglioma. [b]US data exclude embryonal sarcoma.

In many large series, boys with ALL have been found to have a worse prognosis than girls (Miller *et al.*, 1983; Steinhorn & Gloeckler Ries, 1988; Gustafsson *et al.*, 1987). This difference between the sexes was also observed in our data until the most recent period; there had been a proportionately greater improvement in survival rates for boys so that by 1983–85 there was little difference between the sexes in the proportions surviving 5 years. It has previously been reported that the influence of sex on survival is greatest during the first 15 months of complete remission but that boys also suffer an excess of late relapses (Sather *et al.*, 1981). In the present series there was relatively little difference between the sexes in the 1-year and 2-year survival rates. The difference between the sexes in longer-term survival has, however, persisted: among children diagnosed during 1980–82 who had survived 5 years, the mortality during the next 4 years was 10% for boys but only 4% for girls. The outlook for very young children aged under 2 with ALL, and especially for infants diagnosed before the first birthday, has always been poor (Cangir *et al.*, 1975; Miller *et al.*, 1983; Leiper & Chessells, 1986). Although the relatively low survival rates for this age group persisted throughout the study period, they did nevertheless show some improvement. This improvement in survival with more intensive treatment has also been reported from clinical studies (Reaman *et al.*, 1987). Since 1981 the UKALL trials have included larger numbers of very young children, who thus now receive more intensive chemotherapy than they did formerly, though with CNS irradiation postponed until age 2.

Survival has been considerably lower among children with ANLL, although there have been improvements, particularly since the mid 1970s. Children with ANLL who were treated at non-teaching hospitals during 1977–84 had a lower survival rate (Stiller, 1988*a*) and it is likely that the trend towards treatment in specialist centres with widening availability of bone marrow transplantation has contributed to the improved survival rate nationally. Five-year survival rates in the United States SEER registries were substantially higher than in Britain for both 1973–76 (19%) and 1977–80 (25%) (Steinhorn & Gloeckler Ries, 1988).

Hodgkin's disease already had a relatively good prognosis by 1971, and a significant further improvement has since taken place. The survival rate nationally was slightly less than the 94% at 5 years reported from two large oncology centres during 1974–82 (Robinson *et al.*, 1984). During 1970–84, 68 children with Hodgkin's disease were included in the British National Lymphoma Investigation, a study in which most of the patients are adults; the 5-year survival rate for those children was 87% (Makepeace *et al.*, 1987), very similar to that nationally. Survival rates in Britain during 1973–81 were similar to those in the United States (Table VI).

Clinical studies of childhood NHL, have been conducted in Britain by the UKCCSG since 1977 (Mott *et al.*, 1984*a*,*b*). The most marked improvement in the prognosis for NHL occurred from that date onwards. Since survival rates at hospitals outside the UKCCSG have lagged behind those at paediatric oncology centres (Stiller, 1988*a*), much of this improvement can be ascribed to the UKCCSG studies. The survival rate during the 1970s was markedly lower than in the United States (Table VI).

The increase in survival rates from medulloblastoma between 1971 and 1977 has been reported previously (Stiller & Lennox, 1983), and was attributed to improvements in post operative survival and increase in doses of radiotherapy. The trend in survival rates since 1977 has been more modest. The survival rates in the national series are appreciably lower than those reported from large clinical trials (Allen *et al.*, 1986) but the trials did not include patients who died without undergoing surgery or post-operatively. The population-based survival rate was, however, also lower in Britain than in the United States (Table VI) for medulloblastoma. For ependymoma and astrocytoma, rates in the two countries were very similar.

Compared with other diagnostic groups there was an unusually high mortality many years after diagnosis for patients with CNS tumours, with no sign of a plateau in the survival curves. Of the 52 deaths so far recorded among 10-year survivors, death certificates indicated that 36 (71%) were due to recurrent tumour, four (8%) to a second neoplasm and 11 (22%) to other causes. These proportions are very similar to those found among 112 deaths occurring 10–19 years after diagnosis in childen who had CNS tumours diagnosed before 1971 (Hawkins *et al.*, 1990); the causes of death, determined from medical records, were recurrent tumour in 82 (73%), second neoplasm in six (5%) and other causes in 24 (21%).

Survival rates for children with neuroblastoma were still low in comparison with most other childhood cancers by 1983–85, despite the substantial improvements which had taken place, especially since 1980. This was a time which saw intensive research into treatment for neuroblastoma using different combinations of cytotoxic drugs with, in some instances, radiotherapy and/or high dose chemotherapy with autologous bone marrow rescue (Pinkerton, 1990), but no survival rates have been published from these studies for all stages combined. The increase in survival rates nationally might be attributable in part to a rise in the proportion of early stage tumours but this could not be checked in the Registry data. The 2-year survival rate of 32% in Denmark during 1970–80 (Carlsen *et al.*, 1986) was somewhat better than the 24% in Britain during 1971–79, but 5-year survival in the United States was appreciably higher than in either country (Table VI).

Survival rates for retinoblastoma were very high throughout the period and have been documented in greater detail elsewhere (Sanders *et al.*, 1988). The continuing mortality at more than 5 years after diagnosis is due largely to the occurrence of second primary tumours in survivors of bilateral retinoblastoma (Draper *et al.*, 1986; Hawkins, 1989).

Survival rates for Wilms' tumour showed a substantial improvement, particularly in the earlier part of the study period. Throughout 1971–85 there has been a succession of clinical trials and studies of Wilms' tumour in Britain, organised at first by the MRC (MRC, 1978; Morris Jones *et al.*, 1983) In the early 1970s, children included in the first MRC trial had a higher survival rate than eligible children who were not included (Lennox *et al.*, 1979). The category of Wilms' tumour in our series included children with bone-metastasising renal tumour of childhood (BMRTC) and rhabdoid renal tumour. We have identified 25 cases of BMRTC during the study period, nearly all of them from national clinical studies. The 3-year survival rate was 68% overall, and 91% for the 11 children diagnosed during 1980 onwards. These survival rates may well be over estimated as there could have been other, unidentified children with BMRTC outside the trials who may have died. Nevertheless, it is clear that this tumour, which originally had a very poor prognosis, is now eminently treatable. We have also recorded 14 cases of rhabdoid tumour, all but one of them diagnosed during 1979 or later. The survival rate 1 year after diagnosis was only 21%, and only one child has so far survived more than 5 years. This very grave prognosis is similar to that observed in the principal clinical trials (Weeks *et al.*, 1989).

The large increase in survival rates for osteosarcoma during the most recent triennium has been reported previously (Stiller, 1988*b*). The present results, based on a larger number of patients and an extended period of follow-up, confirm the trend noted in that preliminary report. The improvement in prognosis during 1981 onwards was attributable to the much greater improvement for children who were treated at paediatric oncology centres (Stiller, 1988*a*). Some children were entered in clinical trials organised by the Medical Research Council during 1975–81 (MRC, 1986*b*) and EORTC-SIOP during 1978–83 (Burgers *et al.*, 1988). Survival rates for children were not reported separately in either study. In the earlier trial, the 5-year survival rate was 27%, with no significant variation by age; in the latter, where children were stated to have a lower survival rate than adults, the rate for all ages combined was 43%. Thus the 5-year rates for children in both studies were probably similar to the 25–35% in the present series during the same period. In comparison with the United States (Table VI), however, the survival for childhood osteosarcoma in Britain during the 1970s was poor.

The combination of a 5-year survival rate well under 50% and no increase in survival rates during the study period which was found in Ewing's sarcoma occurred for no other diagnostic group. Higher survival rates have been reported for clinical series in Britain (Graham-Pole, 1979). Population-based survival rates in the United States were also somewhat higher than in Britain (Table VI).

The increase in survival rates for rhabdomyosarcoma took place concurrently with large clinical trials in Europe and the United States in which various combinations of chemotherapy and radiotherapy were evaluated. The 5-year survival rates reported here were nevertheless appreciably lower than the 55% in the Intergroup Rhabdomyosarcoma Study (IRS)-I which was open during 1972–78 (Maurer *et al.*, 1988*a*), the 52% in the SIOP trial of 1975–82 (Rodary *et al.*, 1988) and the 62% in IRS-II of 1978–84 (Maurer *et al.*, 1988*b*). The American population-based survival rate was also higher than that in Britain (Table V), but very similar to those in IRS-I (Maurer *et al.*, 1988*a*). The 5-year survival rate for children treated at two large oncology centres in Britain during 1974–81 was 58% (Kingston *et al.*, 1983), higher than in the present series but similar to the American results (Table VI). The variation in survival rate between primary sites, with the best prognosis for tumours of the orbit and the genitourinary sites, was similar to that found in IRS-I and the SIOP trial (Maurer *et al.*, 1988*a*; Rodary *et al.*, 1988).

The improvements in survival for germ-cell tumours were the result of the use of increasingly effective chemotherapy regimes. The role of chemotherapy in ovarian tumours during the first half of the study period has been previously described (La Vecchia *et al.*, 1983). The improvements in more recent years were parallel with those in the UKCCSG germ-cell tumour studies (Mann *et al.*, 1989), in which many of the children analysed here were entered.

A detailed study of children with carcinomas diagnosed during 1971–80 has recently been published (McWhirter *et al.*, 1989). Carcinoma of the thyroid already had an excellent prognosis. The second most common site was the nasopharynx; there was no sign of any increase in survival rates, despite the recently reported improvement in prognosis for children treated with adjuvant chemotherapy in conjunction with radiotherapy (Roper *et al.*, 1986).

The improvements in survival rates for nearly all diagnostic groups described above took place during a period when increasing numbers of chilren were treated at specialist centres participating in national and international clinical trials and studies. It is hoped that this centralisation of care will provide the opportunity for further improvements in the prognosis for many childhood cancers.

We thank the many consultants and general practitioners who provided the information on which this paper is based. We are grateful to the Office of Population Censuses and Surveys, the Information and Statistics Division of the Common Services Agency of the Scottish Health Services, the Registrar General for Scotland, regional cancer registries, clinical trial organisers and the UKCCSG for providing copies of notifications of childhood cancer cases and also to the National Health Service Central Registrars at Southport and Edinburgh for notification of deaths and the flagging of survivors. We thank Professor J.M. Chessells, Dr A.W. Craft and Dr G.J. Draper for helpful comments on earlier drafts. We are grateful to Mr M. Loach and Dr M. Potok for help with computing, to Mrs M. Allen and Dr E.L. Lennox for their part in collecting the medical records and to Mrs E.M. Roberts for secretarial help. The Childhood Cancer Research Group is supported by the Department of Health, the Scottish Home and Health Department and the Cancer Research Campaign.

### References

ALLEN, J., BLOOM, J., ERTEL, I. & 7 others (1986). Brain tumours in children. Current cooperative and institutional chemotherapy in newly diagnosed and recurrent disease. *Semin. Oncol.*, **13**, 110.

BIRCH, J.M. & MARSDEN, H.B. (1987). A classification scheme for childhood cancer. *Int. J. Cancer*, **40**, 620.

BIRCH, J.M., MARSDEN, H.B., MORRIS JONES, P.H., PEARSON, D. & BLAIR, V. (1988). Improvements in survival from childhood cancer: results of a population based survey over 30 years. *Br. Med. J.*, **296**, 1372.

BURGERS, J.M.V., VAN GLABBEKE, M., BUSSON, A. & 13 others (1988). Osteosarcoma of the limbs: report of the EORTC-SIOP 03 trial 20781 investigating the value of adjuvant treatment with chemotherapy and/or prophylactic lung irradiation. *Cancer*, **61**, 1024.

CANGIR, A., GEORGE, S. & SULLIVAN, M. (1975). Unfavorable prognosis of acute leukaemia in infancy. *Cancer*, **36**, 1973.

CARLSEN, N.L.T., JARLE CHRISTENSEN, I.B., SCHROEDER, H. & 5 others (1986). Prognostic factors in neuroblastomas treated in Denmark from 1943 to 1980. A statistical estimate of prognosis based on 253 cases. *Cancer*, **58**, 2726.

CRAFT, A.W., AMINEDDINE, H.A., SCOTT, J.E.S. & WAGGET, J. (1987). The Northern Region Children's Malignant Disease Registry 1968–82: incidence and survival. *Br. J. Cancer*, **56**, 853.

DRAPER, G.J., BIRCH, J.M., BITHELL, J.F. & 6 others (1982). *Childhood Cancer in Britain: Incidence, Survival and Mortality*. Office of Population Censuses & Surveys, Studies on Medical and Population Subjects, no. 37.

DRAPER, G.J., SANDERS, B.M. & KINGSTON, J.E. (1986). Second primary neoplasms in patients with retinoblastoma. *Br. J. Cancer*, **53**, 661.

GRAHAM-POLE, J. (1979). Ewing sarcoma: treatment with high dose radiation and adjuvant chemotherapy. *Med. Pediatr. Oncol.*, **7**, 1.

GUSTAFSSON, G., GARWICZ, S., HERTZ, H. & 8 others (1987). A population-based study of childhood acute lymphoblastic leukemia diagnosed from July 1981 through June 1985 in the five Nordic countries: incidence, patient characteristics and treatment results. *Acta Paediatr. Scand.*, **76**, 781.

HAWKINS, M.M. (1989). Long term survival and cure after childhood cancer. *Arch. Dis. Child.*, **64**, 798.

HAWKINS, M.M., KINGSTON, J.E. & KINNIER WILSON, L.M. (1990). Late deaths following treatment for childhood cancer. *Arch. Dis. Child.* (in the press).

KINGSTON, J.E., MCELWAIN, T.J. & MALPAS, J.S. (1983). Childhood rhabdomyosarcoma: experience of the Children's Solid Tumour Group. *Br. J. Cancer*, **48**, 195.

LA VECCHIA, C., MORRIS, H. & DRAPER, G.J. (1983). Malignant ovarian tumours in childhood in Britain, 1962–78. *Br. J. Cancer*, **48**, 363.

LEIPER, A.D. & CHESSELLS, J. (1986). Acute lymphoblastic leukaemia under 2 years. *Arch. Dis. Child.*, **61**, 1007.

LENNOX, E.L., STILLER, C.A., MORRIS JONES, P.H. & KINNIER WILSON, L.M.(1979). Nephroblastoma: treatment during 1970–73 and the effect on survival of inclusion in the first MRC trial. *Br. Med. J.*, **ii**, 567.

MCWHIRTER, W.R., STILLER, C.A. & LENNOX, E.L. (1989). Carcinomas in childhood: a registry based study of incidence and survival. *Cancer*, **63**, 2242.

MAKEPEACE, A.R., MACLENNAN, K.A., VAUGHAN HUDSON, G. & JELLIFFE, A.M. (1987). Hodgkin's disease in childhood: the British National Lymphoma Investigation experience (BNLI report no. 27). *Clin. Radiol.*, **38**, 7.

MANN, J.R., PEARSON, D., BARRETT, A., RAAFAT, F., BARNES, J.M. & WALLENDSZUS, K.R. (1989). Results of the United Kingdom Children's Cancer Study Group's malignant germ cell tumor studies. *Cancer*, **63**, 1657.

MAURER, H.M., BELTANGADY, M., GEHAN, E.A. & 15 others (1988*a*). The Intergroup Rhabdomyosarcoma Study-1: a final report. *Cancer*, **61**, 209.

MAURER, H.M., GEHAN, E., HAYS, D., NEWTON, W. & TEFFT, M. (1988*b*). Intergroup Rhabdomyosarcoma Study (IRS)-II: a final report (abstract). *Med. Pediatr. Oncol.*, **16**, 414.

MEDICAL RESEARCH COUNCIL (1978). Management of nephroblastoma in childhood. Clinical study of two forms of maintenance therapy. *Arch. Dis. Child.*, **53**, 112.

MEDICAL RESEARCH COUNCIL (1986*a*). Improvement in treatment for children with acute lymphoblastic leukaemia. The Medical Research Council UKALL trials, 1972–84. *Lancet*, **i**, 408.

MEDICAL RESEARCH COUNCIL (1986*b*). A trial of chemotherapy in patients with osteosarcoma (a report to the Medical Research Council by the Working Party on Bone Sarcoma). *Br. J. Cancer*, **53**, 513.

MILLER, D.R., LEIKIN, S., ALBO, V., SATHER, H., KARON, M. & HAMMOND, D. (1983). Prognostic factors and therapy in acute lymphoblastic leukemia of childhood: CCG-141. A report from Children's Cancer Study Group. *Cancer*, **51**, 1041.

MORRIS JONES, P., MARSDEN, H.B. & PEARSON, D. (1983). The conclusion of the 2nd MRC nephroblastoma study (abstract). *Arch. Dis. Child.*, **58**, 653.

MOTT, M.G., CHESSELLS, J.M., WILLOUGHY, M.L. & 4 others (1984*a*). Adjuvant low dose radiation in childhood T cell lymphoma/leukaemia (report from the United Kingdom Children's Cancer Study Group). *Br. J. Cancer*, **50**, 457.

MOTT, M.G., EDEN, O.B. & PALMER, M.K. (1984*b*). Adjuvant low dose radiation in childhood non-Hodgkin's lymphoma (report from the United Kingdom Children's Cancer Study Group). *Br. J. Cancer*, **50**, 463.

PAOLUCCI, G., MASERA, G., VECCHI, V., MARSONI, S., PESSION, A. & ZURLO, M.G. (1989). Treating childhood acute lymphoblastic leukaemia (ALL): summary of ten years' experience in Italy. *Med. Pediatr. Oncol.*, **17**, 83.

PARKIN, D.M., STILLER, C.A., DRAPER, G.J., BIEBER, C.A., TERRACINI, B. & YOUNG, J.L. (eds) (1988). *International Incidence of Childhood Cancer*. IARC Scientific Publications, no. 87: Lyon.

PINKERTON, C.R. (1990). Where next with therapy in advanced neuroblastoma? *Br. J. Cancer*, **61**, 351.

POLEDNAK, A.P. (1986). Recent trends in incidence and mortality rates for leukemias, and in survival rates for childhood acute lymphocytic leukemia, in upstate New York. *Cancer*, **57**, 1850.

REAMAN, G.H., STEINHERZ, P.G., GAYNON, P.S. & 6 others (1987). Improved survival of infants less than 1 year of age with acute lymphoblastic leukaemia treated with intensive multiagent chemotherapy. *Cancer Treat. Rep.*, **71**, 1033.

ROBINSON, B., KINGSTON, J., NOGUEIRA COSTA, R., MALPAS, J.S., BARRETT, A. & MCELWAIN, T.J. (1984). Chemotherapy and irradiation in childhood Hodgkin's disease. *Arch. Dis. Child.*, **59**, 1162.

RODARY, C., REY, A., REZVANI, A. & FLAMANT, F. (1988). Facteurs prognostiques des rhabdomyosarcomes de l'enfant: etude realisee sur 253 enfants enregistres par la Societe Internationale d'Oncologie Pediatrique (SIOP). *Bull. Cancer*, **75**, 213.

ROPER, H.P., ESSEX-CATER, A., MARSDEN, H.B., DIXON, P.F. & CAMPBELL, R.H.A. (1986). Nasopharyngeal carcinoma in children. *Pediatr. Hematol. Oncol.*, **3**, 143.

SANDERS, B.M., DRAPER, G.J. & KINGSTON, J.E. (1988). Retinoblastoma in Britain 1969–80: incidence, treatment and survival. *Br. J. Ophthalmol.*, **72**, 576.

SATHER, H., COCCIA, P., NESBIT, M., LEVEL, C. & HAMMOND, D. (1981). Disappearance of the predictive value of prognostic variables in childhood acute lymphoblastic leukemia: a report from Children's Cancer Study Group. *Cancer*, **48**, 370.

STEINHORN, S.C. & GLOECKLER RIES, L. (1988). Improved survival among children with acute leukemia in the United States. *Biomed. Pharmacother.*, **42**, 675.

STILLER, C.A. (1988*a*). Centralisation of treatment and survival rates for cancer. *Arch. Dis. Child.*, **63**, 23.

STILLER, C.A. (1988*b*). Treatment of osteosarcoma. *Lancet*, **i**, 931.

STILLER, C.A. & DRAPER, G.J. (1989). Treatment centre size, entry to trials and survival in acute lymphoblastic leukaemia. *Arch. Dis. Child.*, **64**, 657.

STILLER, C.A. & LENNOX, E.L. (1983). Childhood medulloblastoma in Britain 1971–77: analysis of treatment and survival. *Br. J. Cancer*, **48**, 835.

WEEKS, D.A., BECKWITH, J.B., MIERAU, G.W. & LUCKEY, D.W. (1989). Rhabdoid tumor of kidney: a report of 111 cases from the National Wilms' tumor study pathology center. *Am. J. Surg. Pathol.*, **13**, 459.

YOUNG, J.L., GLOECKLER RIES, L., SILVERBERG, E., HORM, J.W. & MILLER, R.W. (1986). Cancer incidence, survival and mortality for children younger than age 15 years. *Cancer*, **58**, 598.